# A new pH sensor localized in the Golgi apparatus of *Saccharomyces cerevisiae* reveals unexpected roles of Vph1p and Stv1p isoforms

**DOI:** 10.1038/s41598-020-58795-w

**Published:** 2020-02-05

**Authors:** Antoine Deschamps, Anne-Sophie Colinet, Olga Zimmermannova, Hana Sychrova, Pierre Morsomme

**Affiliations:** 1 Louvain Institute of Biomolecular Science and Technology, Université catholique de Louvain, B-1348 Louvain-la-Neuve, Belgium; 2 Institute of Physiology, Czech Academy of Sciences, CZ-14220 Prague, Czech Republic

**Keywords:** Sensors and probes, Organelles, Golgi, Fungi, Microbiology

## Abstract

The gradual acidification of the secretory pathway is conserved and extremely important for eukaryotic cells, but until now there was no pH sensor available to monitor the pH of the early Golgi apparatus in *Saccharomyces cerevisiae*. Therefore, we developed a pHluorin-based sensor for *in vivo* measurements in the lumen of the Golgi. By using this new tool we show that the *cis*- and *medial*-Golgi pH is equal to 6.6–6.7 in wild type cells during exponential phase. As expected, V-ATPase inactivation results in a near neutral Golgi pH. We also uncover that surprisingly Vph1p isoform of the V-ATPase is prevalent to Stv1p for Golgi acidification. Additionally, we observe that during changes of the cytosolic pH, the Golgi pH is kept relatively stable, mainly thanks to the V-ATPase. Eventually, this new probe will allow to better understand the mechanisms involved in the acidification and the pH control within the secretory pathway.

## Introduction

An acute regulation of the intracellular pH is particularly important for most of the biological processes because protein structures as well as enzyme activities rely on this parameter^1^. In the secretory pathway, the pH is becoming gradually more acidic from the endoplasmic reticulum (ER) to secretory vesicles or to the vacuole/lysosomes. This gradual acidification is crucial to trigger the activation of some enzymes involved in post-translational modifications and degradation processes. For example, it is the case for many proteases which are turned *“ON”* when they reach the acidic vacuole^2,3^. Similarly, some glycosidases and glycosyltransferases become active once they face the appropriate pH, in a specific compartment of the secretory pathway^4^. In addition, the pH also controls the trafficking and localization of these enzymes within the secretory pathway^5^. Many receptors have pH-dependent affinity for their ligand. It is particularly well described for several plasma membrane receptors which bind to their target at the plasma membrane and dissociate once the pH drops in endosomes^6^, for the delivery of lysosomal proteases to their destination thanks to the mannose-6-phosphate receptor^7^, or for the retrieval of ER-resident proteins that are recycled from the Golgi to the ER thanks to the KDEL receptor^8,9^. Furthermore, the pH gradient across biological membranes serves as the driving force for many secondary transporters. While at the plasma membranes the nature of this electrochemical gradient differs between the different kingdoms of life, the pH gradient is the main electrochemical gradient used in organelles of all eukaryotes by secondary transporters. The vacuolar H^+^-ATPase (V-ATPase) is the main pump responsible for the acidification of the secretory pathway and the electrochemical balance is controlled by a Golgi pH regulator which is an anion channel^10^, probably in collaboration with a still unidentified proton leak channel^11^. When these acidification mechanisms are not perfectly functional at the Golgi level, it may lead to various diseases such as congenital disorders of glycosylation, *Cutis laxa* or non-syndromic intellectual disability^12–15^.

Given the importance of pH homeostasis within the cell and the secretory pathway (reviewed in Casey *et al*.^16^), it is essential to possess appropriate tools to accurately measure this parameter *in vivo*. For the yeast *S*. *cerevisiae*, one probe is already available to measure the pH of the *trans*-Golgi network/endosomes lumen^17,18^ and the chemical probe BCECF is commonly used to measure the vacuolar pH^19^. Recently, another sensor has been developed for pH measurements within the ER^20^. However, there is no sensor suitable to measure precisely the early Golgi pH. For this reason, we engineered a pH probe for the Golgi lumen. With this tool, we reveal that Vph1p plays a prevalent role compared to Stv1p for the sustenance of an acidic Golgi lumen and that the Golgi pH is actively kept acidic when the cytosolic pH fluctuates, mainly due to the V-ATPase. In the future, this new probe will allow to better understand how the acidification occurs and is controlled in the Golgi apparatus. It could also give guidelines to design other probes very specifically targeted to the different steps of the secretory pathway.

## Results and Discussion

### Targeting of an improved pHluorin-based probe to the Golgi apparatus of *S*. *cerevisiae*

**Figure 1:**
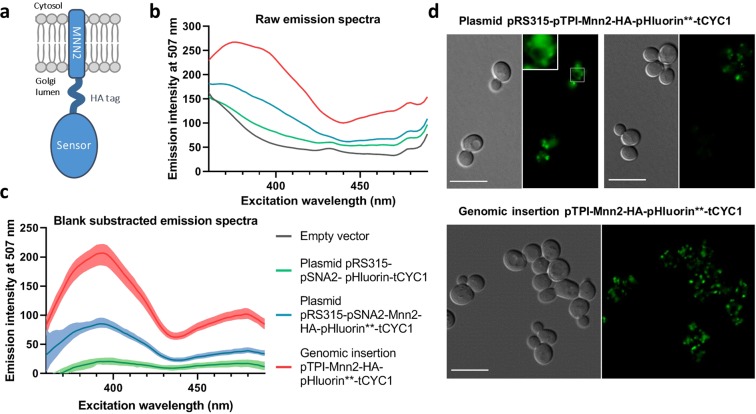
A pHluorin-based pH sensor targeted to the Golgi apparatus was designed and step by step optimized. (**a**) Expected topology of the chimeric protein. The 36 first amino acids of the Mnn2 protein contain a predicted type-II transmembrane domain and aims at targeting the pHluorin probe to the Golgi lumen. The transmembrane part and the sensor part are linked by 3Gly-HAtag-3Gly sequence which also serves as a spacer. (**b**) Adaptations to enhance the fluorescence intensity of the Golgi pH probe. All measurements were done with the BY4742 wild type background strain, transformed either with an empty plasmid (Empty vector, gray curve), with a plasmid containing the original pHluorin under the control of the weak pSNA2 promoter (pRS315-pSNA2-Mnn2-HA-pHluorin-tCYC1, green curve), with the same vector containing the pHluorin with two punctual mutations – F64L and M153R – (pRS315-pSNA2-Mnn2-HA-pHluorin**-tCYC1, blue curve), or with the genomic insertion of the double mutated pHluorin under the control of the strong constitutive pTPI promoter (Genomic insertion pTPI-Mnn2-HA-pHluorin**-tCYC1, red curve). (**c**) Blank subtracted spectra reveal the specific fluorescence of the probe. The fluorescent background of wild type cells transformed with an empty plasmid was subtracted to raw fluorescent spectra represented in part (b). *N* = 3. Means and ranges are represented. (**d**) Genomic integration improves homogeneity of the expression level of the protein of interest, compared to classical plasmid expression system. Fluorescence microscopy images of cells expressing Mnn2-HA-pHluorin** on a plasmid (upper part) or inserted within the genome (lower part), both under the control of the pTPI promoter. Left: DIC; right: GFP filter. Scale bars = 10 µm.

Proteins are precisely localized within one or another organelle thanks to various targeting signals and retention mechanisms. We took advantage from the efficient retention mechanisms of Golgi-localized proteins to address the pHluorin – a ratiometric pH probe derived from GFP^21^ – to the *cis*- and *medial*-Golgi apparatus. For this, we fused it with the transmembrane span of Mnn2p, an alpha-1,2-mannosyltransferase localized in the early Golgi^22^. The first 36 amino acids of Mnn2p^23^ have been linked to the N-terminus of the pHluorin via a HA tag which serves as a spacer to avoid noxious interactions of the pH probe with the phospholipidic membrane (Fig. 1a). Considering that the Golgi apparatus represents only a small fraction of the total cellular volume, the expression level of Golgi-localized proteins has to be maintained at a relatively low level to avoid mistargeting. Therefore, we expressed the construct on a pRS315 plasmid under the control of pSNA2, the weak constitutive promoter of the SNA2 gene^24^. Unfortunately, the resulting fluorescence of our probe was very close to the fluorescent background (Fig. 1b). Interestingly, several groups have identified punctual mutations that increase the brightness of the pHluorin with regards to the original version^25,26^. We inserted two of these mutations, F64L and M153R, respectively, to generate the Mnn2-HA-pHluorin** chimeric protein. This strategy enhances the specific fluorescent signal of about 300% (Fig. 1b,c). Moreover, using a protease degradation assay, we observed that the mutated form of the pHluorin is much more resistant to protease degradation than the original version (data not shown), indicating that the stability of the protein could be increased thanks to these two mutations. According to Reifenrath *et al*.^20^ that recently developed a pHluorin variant to measure the pH within the ER of *S*. *cerevisiae*, these mutations could facilitate the folding of the protein in the oxidative environment of the ER lumen.

By fluorescence microscopy, we could detect the probe in punctuated dots in many cells, which is the typical Golgi shape in *S*. *cerevisiae*. However, in some cells, it also exhibits the classical ER-like pattern or it stains the vacuoles (Fig. 1d, upper panel). Given the heterogeneity of the signal, we inserted the construct within the genome, as it is known to homogenize the expression level of a protein of interest from cell to cell^27^. This strategy highly improved the homogeneity of the localization (Fig. 1d, lower panel) and therefore the robustness of the next measurements, but the fluorescent level was still weak. Our last improvement consisted of changing the promoter to enhance the fluorescent signal. We replaced the pSNA2 promoter by a stronger constitutive promoter, namely pTPI. Compared to the plasmid expression system with pSNA2 promoter, these two last modifications increased the total fluorescent signal by a 2.5 fold, facilitating future pH determination (Fig. 1b,c). For all the subsequent experiments in this study, we used the genome integrated pTPI-Mnn2-HA-pHluorin** strains. In addition, when doing fluorescence measurements, we always subtracted the fluorescence background, which stands for 20 to 30% of the total fluorescent signal, as measured with cells transformed with empty plasmid (Fig. 1b).

### The pH sensor localizes in the lumen of the *cis-* and *medial-*Golgi

**Figure 2:**
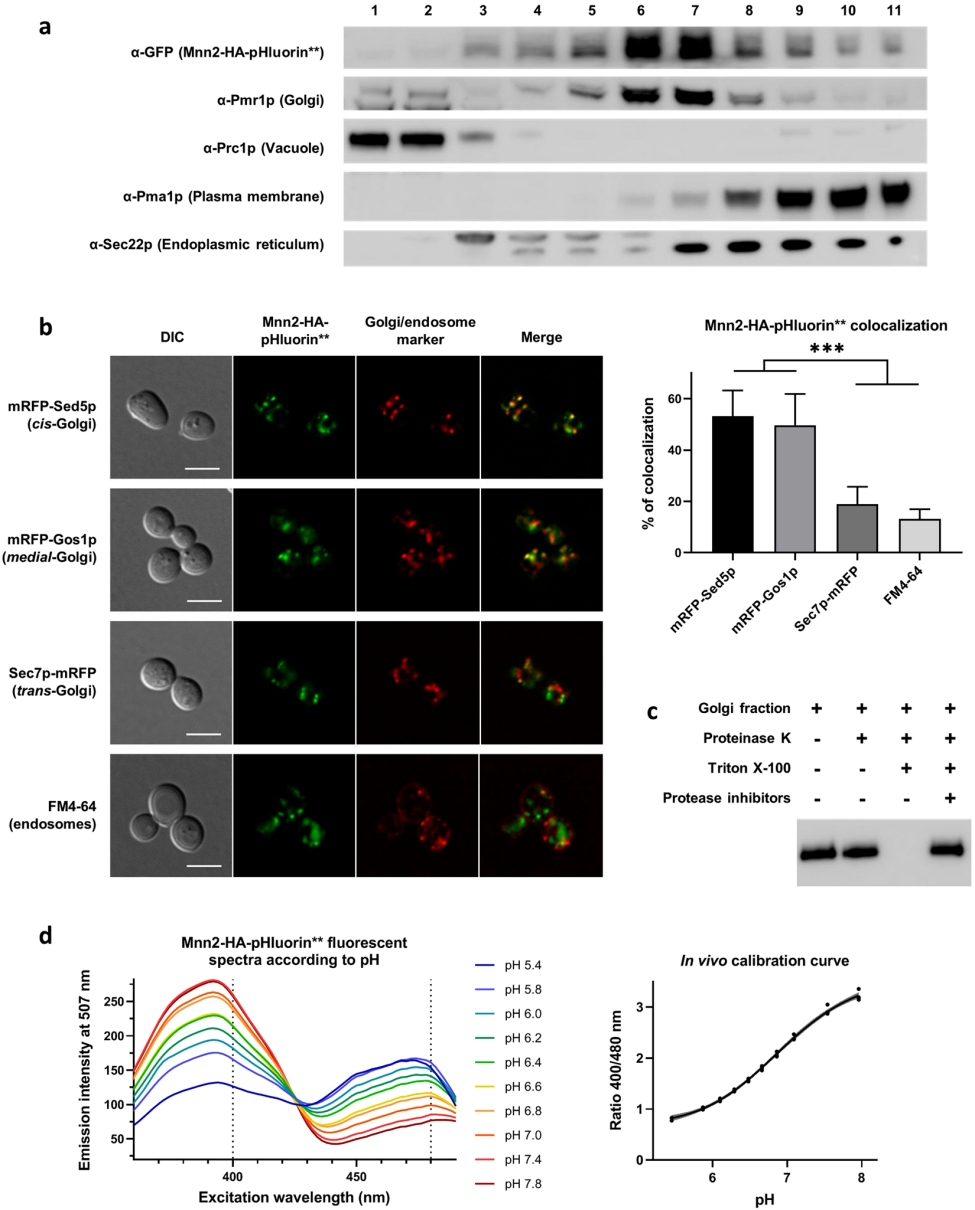
The pH sensor is localized in the *cis*- and *medial*-Golgi and correctly responds to pH *in vivo*. (**a**) According to subcellular fractionation, the pH probe co-fractionates with Golgi marker. Cells expressing the genome integrated Mnn2-HA-pHluorin** construct where fractionated on a discontinuous 12.5–54% sucrose gradient. After ultracentrifugation, the fractions were collected and numbered from the top of the gradient and were analyzed by Western blot using antibodies against the sensor (α-GFP) or against Pmr1p (Golgi apparatus), Pma1p (Plasma membrane), Sec22p (Endoplasmic reticulum), or Prc1p (Vacuole). (**b**) The Mnn2-HA-pHluorin** protein co-localizes mainly with *cis*- and *medial*-Golgi markers. Different Golgi markers were assessed by fluorescence microscopy – mRFP-Sed5p (*cis*-Golgi), mRFP-Gos1p (*medial*-Golgi), Sec7p-mRFP (*trans*-Golgi) – and FM4–64 chemical dye was used for endosomes detection. Left part: representative images of cells imaged by DIC, with GFP filter, with RFP filter, and merge of the two fluorescent channels. Right part: co-localization was quantified as the number of Mnn2-HA-pHluorin** positive structures that co-localizes with these markers compared to the total number of Mnn2-HA-pHluorin** positive structures, using an object-based approach (*N* = 12 pictures, 31–38 cells per condition, except for FM4-64 for which *n* = 8 pictures, 101 cells). Scale bars = 5 µm. Means and 95% confidence intervals are shown. (**c**) A topology assay attests that the probe is facing the lumen of the Golgi apparatus. Golgi-enriched fractions obtained by subcellular fractionation on sucrose gradient were submitted to 1 µg/ml proteinase K during 1 hour at 30 °C. The first lane is the positive control corresponding to the input; the second lane is the topology test, wherein the Golgi vesicles are incubated with proteinase K; the third lane is a negative control, the Golgi vesicles being incubated simultaneously with proteinase K and 1% Triton X-100 to permeabilize the phospholipidic membranes; the fourth lane corresponds to the negative control to which PMSF and a mix of protease inhibitors have been added. Anti-HA primary antibodies were used. (**d**) *In vivo* calibration of the probe was performed. Cells expressing the sensor were permeabilized with 0.16% digitonin, followed by an incubation in citric acid – sodium hydrogen phosphate buffers at different pH, and their excitation spectra were measured with emission at 507 nm. Left part: the different excitation spectra of cells in pH buffers ranging from pH 5.4 to 7.8 are represented. Right part: calibration curve of the pH versus 400/480 nm excitation ratio. A four-parameter logistical curve (sigmoidal curve) has been drawn through the experimental measurements. *N* = 3–4. Calibration curve is represented with 99% confidence interval.

To further confirm that the probe is correctly localized at the Golgi apparatus, we carried out subcellular fractionations on sucrose gradient and performed co-localization fluorescence microscopy with Golgi and endosomal markers. The fractionation pattern of the Mnn2-HA-pHluorin** protein perfectly fits the one of Pmr1p, a Golgi resident protein. In contrast, the analysis of vacuolar, endoplasmic reticulum and plasma membrane markers demonstrate a distinct distribution profile for these organelles (Fig. 2a). Then, Sed5p, Gos1p and Sec7p were respectively used as markers of the *cis*-, *medial*- and *trans*-Golgi for fluorescence microscopy^28^. Endosomes detection relies on the endocytic marker FM4-64. We observed that the Mnn2-HA-pHluorin** construct mainly co-localizes with *cis*- and *medial*-Golgi markers and that a small fraction of the probe presumably also localizes further in the secretory pathway, within the *trans-*Golgi and the endosomes (Fig. 2b). Quantification of the co-localization was performed on fixed cells using an object-based methodology (Fig. 2b, right part). For endosomal detection with FM4-64, the quantifications performed on fixed cells and with living cells gave very similar results (13.2% of co-localization with fixed cells, 13.3% of co-localization with living cells). Therefore, only the quantification with fixed cells is represented in Fig. 2b. Overall, the two localization experiments endorse that the chimeric Mnn2-HA-pHluorin** protein localizes to the Golgi apparatus.

As a second step, we verified that the sensor is correctly oriented towards the lumen of the organelle, taking advantage of a protease degradation assay. After subcellular fractionation on sucrose gradient, Golgi-enriched fractions were incubated with proteinase K during 1 hour at 30 °C (Fig. 2c, 2^nd^ lane). In parallel, an equivalent sample was incubated concomitantly with proteinase K and Triton X-100 to permeabilize the Golgi phospholipidic membrane (Fig. 2c, 3^rd^ lane). After the digestion was stopped with a mixture of protease inhibitors, these samples were analyzed by *western blot*, compared together, as well as to the input (Fig. 2c, 1^st^ lane) and to a control of inhibitors efficiency (Fig. 2c, 4^th^ lane). Indubitably, the pHluorin part is protected from proteinase K digestion by the Golgi lipid bilayer. Thereby, the sensor properly faces the Golgi lumen.

### *In vivo* calibration and determination of the Golgi pH

The original pHluorin responds to the surrounding pH in a range from 5.5 to 8.0^21^. Despite the fact that the addition of the two mutations (F64L and M153R) separately does not strongly alter the pH-sensitive properties of the probe^25,26^, the combined addition of the two mutations could potentially distort the functionality of the sensor. Therefore, we performed an *in vivo* calibration of the probe by resuspending the cells in various pH buffers after permeabilization of both the plasma membrane and the Golgi membrane with 0.16% digitonin. By doing so, the blank corrected fluorescent spectra of the Mnn2-HA-pHluorin** protein perfectly responds to the surrounding pH, with opposite effects on the excitation at 400 or 480 nm when the pH fluctuates (Fig. 2d, left panel). By using the fluorescent ratio of emission at 507 nm after excitation at 400 and 480 nm and plotting it versus pH, the calibration is obtained (Fig. 2d, right panel). The sensor is therefore suitable for *in vivo* determination of the pH within the Golgi lumen.

**Figure 3:**
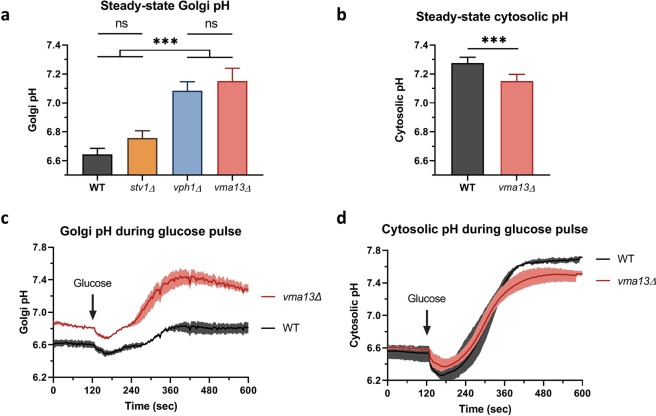
Golgi and cytosolic pH measurements at steady-state and during glucose pulse. Steady-state Golgi (**a**) and cytosolic (**b**) pH measurements of cells grown in synthetic medium. Cells were collected during exponential growth phase, resuspended in fresh medium and directly transferred into the fluorimeter for measurement. The fluorescent measurements were then converted into pH values thanks to *in vivo* pH calibration. *N* = 4–9. Means and 95% confidence intervals are represented. Golgi (**c**) and cytosolic (**d**) pH measurements of cells starved of glucose during 30 min, to which glucose is added back. Cells were collected during exponential growth phase, washed, resuspended into synthetic medium lacking glucose and incubated during 30 min on ice. Cells were then incubated during 5 min at 28 °C prior to fluorescence measurement. After 120 sec of recording, glucose was added to the cell suspension at a final concentration of 2% and the measurement was prolonged up to 600 sec. The fluorescence was recorded with alternate excitation at 400 and 480 nm and the resultant fluorescent ratio was converted into pH value. *N* = 3 for Golgi pH measurements, *N* = 5 for cytosol pH measurements. Means and ranges are represented.

Cytosolic and Golgi pH measurements were performed in parallel (Fig. 3a,b) using a cytosolic pHluorin^29^ and our newly developed Golgi-localized probe. As expected, the Golgi pH of cells in exponential phase is more acidic than the cytosolic pH, with a pH value of 6.65 ± 0.05 for the Golgi lumen, while the cytosolic pH is 7.27 ± 0.05. This is consistent with the expected Golgi pH value^16,30^ and with some measurements performed in other organisms, such as Tobacco and *A*. *thaliana* plants^31,32^ and mammalian cells^33,34^. This value for the Golgi pH is consistent with the gradual acidification of the secretory pathway. Indeed, endoplasmic reticulum pH and vacuolar pH of *S*. *cerevisiae* cells fed with glucose in exponential phase are equal to 7.1 and ≤6.0, respectively^20,35,36^.

### The V-ATPase is the main H^+^-transporter of the early Golgi and Vph1p isoform has predominant activity compared to Stv1p isoform

We measured the pH of the early Golgi in several strains deleted for different subunits of the V-ATPase. In the *vma13*Δ strain, Vma13p being the H subunit of the V-ATPase, the Golgi lumen becomes near neutral (Fig. 3a). Indeed, the V-ATPase has completely lost its activity in this strain. This result supports that the V-ATPase is the main proton pump in charge of the acidification of the secretory pathway. It also ensures that the pH sensor is functional and correctly oriented towards the Golgi lumen. Simultaneously, the cytosolic pH exhibits the opposite trend, being more acidic in the absence of a functional V-ATPase – as measured in the *vma13*Δ strain – than in the wild type strain (Fig. 3b)^35^. In yeast, all the subunits of the V-ATPase have only one isoform, except the “a” subunit of the V_0_ domain which exists as two isoforms. Vph1p mainly localizes in the vacuole, while Stv1p is found in V-ATPase complexes of the Golgi apparatus and of endosomes^37–40^. Using the new Golgi pH probe, we show that deletion of *STV1* only slightly increases the Golgi pH (Fig. 3a). This corroborates phenotypic assays, protein sorting and glycosylation analysis performed previously^38,41,42^. One explanation would be that the second isoform, Vph1p, that is several folds more expressed than Stv1p^38,43^, is sufficiently efficient to acidify the Golgi and the endosomes during its transit *en route* to the vacuole. In contrast, the deletion of *VPH1* strongly increases the Golgi pH compared to the wild type strain, almost to the same level as the *vma13*Δ strain (Fig. 3a). It suggests that Vph1p plays a major role for the acidification of the Golgi apparatus and it contradicts the classical model of Stv1p being the main V-ATPase “a” subunit in the Golgi apparatus.

### The V-ATPase moderates pH fluctuations in the Golgi during a glucose pulse

It is well described that glucose availability influences the cytosolic pH^44–46^. Especially, when cells are starved of glucose, the cytosolic pH decreases to a considerably lower value. Added back in the external medium, the glucose is transported into the cell, glycolysis is activated, ATP is produced and the proton pumps will extrude protons out of the cytosol^35,47^. As expected, when we monitored the cytosolic pH of glucose starved cells during glucose re-addition, we observed first a transient extra acidification – presumably due to glycolysis which produces protons – followed by an alkalinization to return to a near neutral pH value. This trend occurs similarly in the wild type and in the *vma13*Δ strain (Fig. 3d), except that the final pH is slightly more acidic in the *vma13*Δ strain, consistently with the steady-state pH measurements. If we monitor the Golgi pH during the same process, the curve follows the same trend, first with a transient acidification that appears a few seconds after glucose addition, followed by a pH increase (Fig. 3c). We should stress the point that the amplitude of pH increase is much lower in the Golgi than in the cytosol. In the wild type strain, the Golgi pH only varies of 0.2 pH unit, while it varies of more than 1.2 pH unit in the cytosol during the same glucose stress. It can be explained by the activity of the V-ATPase which pumps protons towards the Golgi lumen. Moreover, it has been previously described that the Golgi V-ATPase complexes does not undergo dissociation in response to glucose starvation, on the contrary to the vacuolar V-ATPase complexes^48^. Accordingly, the Golgi pH is kept relatively stable and does not experience the same alkalinization as the cytosolic pH after glucose re-addition. In comparison, the Golgi pH of *vma13*Δ varies of 0.6 pH unit, while the cytosolic pH of the same strain varies of about 1.0 pH unit. Altogether, it shows that the Golgi pH is affected by its cytosolic counterpart, possibly due to weak acids and/or to proton channels and proton exchangers, although being able to “smooth” the variations compared to what is happening in the surrounding cytosol. In addition, it confirms that the V-ATPase is essential to continuously maintain acidic the Golgi pH, because its absence makes the Golgi much more responsive to cytosolic pH variations.

### Conclusion and outlook

In this study, we developed a Golgi-localized pH sensor for *S*. *cerevisiae* based on the genetically encoded pHluorin^21^. Its fluorescence level was increased by means of some specific mutations, making it suitable for measurements of a small organelle such as the Golgi apparatus. In our strategy, we fused the probe with the single transmembrane span of a type II membrane protein, which is very frequent for many glycosylation enzymes. This methodology was efficient to precisely target the chimeric protein at the *cis*- and *medial*-Golgi with the right topology, the probe facing the Golgi lumen.

Using our newly developed Golgi probe, we observed that the Golgi pH is surprisingly much more affected by the deletion of *VPH1* than by the deletion of *STV1*. As Stv1p is commonly described as the “a” subunit isoform of the V-ATPase localized in the Golgi and the endomembrane system while Vph1p reaches the vacuolar membrane, the opposite trend was expected^38^. Of course, Vph1p transits via the secretory pathway to reach the vacuole and transiently co-localizes with Stv1p^49^ and it has been previously suggested that Vph1p could already form a functional V-ATPase involved in the acidification of the Golgi apparatus^18^. Here, our data highlight that Vph1p is actually the main player for the acidification of the Golgi lumen while Stv1p plays only a minor role, at least when cells are exponentially growing. Stv1p and Vph1p derive from a common ancestor gene that was probably able to fulfill V-ATPase roles both at the Golgi and in the vacuole^50^, as it is also the case for some actual species that possess one single “a” subunit^51^. Therefore, Stv1p and Vph1p isoforms emergence during evolution probably gave a selective fitness advantage to actual budding yeast cells compared to their ancestor with a single “a” subunit. Keeping Vph1p involved in the usual acidification of the secretory pathway allowed maybe Stv1p to take other more specialized roles, like pH sensing, membrane fusion or V-ATPase regulation^52–54^.

Another interesting observation is that, while the cytosolic pH is strongly affected by glucose availability, the Golgi pH is kept quite stable regardless glucose concentration in the external medium. Moreover, this Golgi pH maintenance is highly due to the presence of the V-ATPase. It is known that vacuolar localized V-ATPase complexes undergo dissociation after glucose deprivation, while Golgi and endosomal Stv1p-containing V-ATPase complexes do not dissociate^39,48,49^. Therefore, V-ATPase complexes specifically located in the Golgi apparatus could promptly pump protons towards the Golgi lumen after glucose re-addition. As a result, the secretory pathway functionalities would be very quickly recovered.

In the future, the strategy we used to develop this sensor could be used to target pH probes to the *trans*-Golgi or the *trans*-Golgi network in order to have a full kit of tools to measure the pH of the secretory pathway. Indeed, there are still many questions regarding how the pH homeostasis is regulated in the late Golgi and the endosomal network, in which the interplay of V-ATPase activity and other mechanisms involved in organellar acidification appears less clear. Notably, *vma* mutants display more acidic Gef1p-containing organelles than wild type cells^17,18^, suggesting that compensating systems exist and/or that the regulation processes are disturbed in these mutants. As of now, our *cis*- and *medial*-Golgi pH probe could maybe sustain the discovery of other H^+^ channels and transporters, as the “H^+^ leak channel” whose identity is still unclear^11,55^. Several membrane proteins are identified as candidates to play this role, being predicted to transport protons through the Golgi and the endosome membranes. It is the case of Gdt1p, a putative Ca^2+^-Mn^2+^/H^+^ antiporter^56,57^, Gef1p, a presumed Cl^−^/H^+^ exchanger^17^, or Nhx1p and Kha1p, predicted sodium and potassium - proton exchangers^58,59^. In conclusion, additional *in vivo* pH measurements would allow a better understanding of the secretory pathway pH regulation. We could decipher how and when the V-ATPase is activated or disassembled, how Vph1p and Stv1p isoforms interplay, or which are the other proteins and mechanisms involved in the regulation of the Golgi pH.

## Material and Methods

### Plasmids and strains

Plasmids were obtained following standard molecular biology protocols and were validated by sequencing. They are listed in Table 1. All the plasmids constructed for this study were made in the pRS315 scaffold. The promoter was cloned between SacI and NotI restriction sites, the Mnn2-HA-pHluorin chimeric sequence was obtained by triple PCR and inserted between NotI and SpeI restriction sites, and the tCYC1 (cytochrome C) terminator inserted between SpeI and HindIII restriction sites. Two punctual mutations within the pHluorin were inserted sequentially by QuickChange PCR. All the primers are listed in Table 2. For genomic insertion, a PCR product of approximately 4.6 kb containing the pTPI-Mnn2-HA-pHluorin**-tCYC1 sequence and the LEU2 gene was generated from the pRS315-pTPI-Mnn2-HA-pHluorin(F64L;M153R)-tCYC1 plasmid using appropriate primers containing flanking sequences corresponding to the his3 locus (primers IntLocHis3LEU2_Fw and IntLocHis3HindIII_Rv). The PCR product was then purified on agarose gel and used for classical yeast transformation according to previously described method^60^, with the addition of 10% DMSO before transferring the cells at 42 °C. Genomic integrations were verified by PCR.

**Table 1:** Plasmids used in this study.

Plasmid	Abbreviate construct name	Source
pRS315-pSNA2-Mnn2-HA-pHluorin-tCYC1	pSNA2-Mnn2-HA-pHluorin	This study
pRS315-pTPI-Mnn2-HA-pHluorin(F64L;M153R)-tCYC1	pTPI-Mnn2-HA-pHluorin**	This study
pRS315-pSNA2-Mnn2-HA-pHluorin(F64L;M153R)-tCYC1	pSNA2-Mnn2-HA-pHluorin**	This study
pRS315-pTPI-USER-tCYC1	Empty vector	Our laboratory
pRS316-mRFP-Sed5	mRFP-Sed5	Matsuura-Tokita *et al*.^28^
pRS316-mRFP-Gos1	mRFP-Gos1	Matsuura-Tokita *et al*.^28^
pRS316-Sec7-mRFP	Sec7-mRFP	Matsuura-Tokita *et al*.^28^
pZR4.1-pTEF1-pHluorin-tCYC1	pHluorin	Gift from R. Rao (Baltimore, MD, USA)^29^

**Table 2:** Primers used in this study.

Primer name	Primer sequence
NotI-MNN2_Fw	5′-CATGCGGCCGCATGCTGCTTACCAAAAGG-3′
MNN2-HA_Rv	5′-ACCTCCGCCGGCGTAGTCTGGGACATCGTATGGGTAACCGCCTCCCGACGTGTTCTCATCCAT-3′
HA-pHluorin_Fw	5′-GGAGGCGGTTACCCATACGATGTCCCAGACTACGCCGGCGGAGGTAGTAAAGGAGAACTTTTCACT-3′
pHluorin-SpeI_Rv	5′-CGGACTAGTTTATTTGTATAGTTCATCCAT-3′
pHluorin-F64L_Fw	5′-GGCCAACACTTGTCACTACTTTATCTTATGGTGTTCAATG-3′
pHluorin-F64L_Rv	5′-CATTGAACACCATAAGATAAAGTAGTGACAAGTGTTGGCC-3′
pHluorin-M153R_Fw	5′-TAACGAGCACTTGGTGTACATCAGGGCAGACAAACA-3′
pHluorin-M153R_Rv	5′- TGTTTGTCTGCCCTGATGTACACCAAGTGCTCGTTA-3′
IntLocHis3LEU2_Fw	5′-GCAGAAAGCCCTAGTAAAGCGTATTACAAATGAAACCAAGCCTTATCACGTTGAGCCATTAG-3′
IntLocHis3HindIII_Rv	5′-CCATTGGGCGAGGTGGCTTCTCTTATGGCAACCGCAAGAGGGTCGACGGTATCGATAAGCTT-3′

The *S*. *cerevisiae* strains used in this study are listed in Table 3.

**Table 3:** Yeast strains used in this study.

Strain	Description	Source
BY4742 wild type	*Mata his3*Δ*1 leu2*Δ0 *met15*Δ0 *ura3*Δ*0*	Euroscarf
BY4742 Mnn2-HA-pHluorin**	*Mata his3*Δ1::LEU2-pTPI-MNN2-HA-pHluorin**-tCYC1 leu2Δ*0 met15*Δ*0 ura3*Δ*0*	This study
BY4742 *vma13*Δ	*Mata his3*Δ*1 leu2*Δ*0 met15*Δ*0 ura3*Δ*0 vma13::KanMX4*	Euroscarf
BY4742 *vma13*Δ Mnn2-HA-pHluorin**	*Mata his3*Δ1::LEU2-pTPI-MNN2-HA-pHluorin**-tCYC1 leu2Δ*0 met15*Δ*0 ura3*Δ*0 vma13::KanMX4*	This study
BY4742 *vph1*Δ Mnn2-HA-pHluorin**	*Mata his3*Δ1::LEU2-pTPI-MNN2-HA-pHluorin**-tCYC1 leu2Δ*0 met15*Δ*0 ura3*Δ*0 vph1::KanMX4*	This study
BY4742 *stv1*Δ Mnn2-HA-pHluorin**	*Mata his3*Δ1::LEU2-pTPI-MNN2-HA-pHluorin**-tCYC1 leu2Δ*0 met15*Δ*0 ura3*Δ*0 stv1::KanMX4*	This study

### Culture conditions

Non-transformed yeast cells were routinely cultured at 28 °C in YD medium (2% yeast extract KAT, 2% glucose) under agitation. Cells transformed with plasmids were grown at 28 °C in SD minimal medium (0.7% yeast nitrogen base without amino acids (Difco), 2% glucose, supplemented with all amino acids except these used as a selection marker for plasmid maintenance) with agitation. Solid media were produced by addition of 2% agar to the mixture. For genomic insertion, after selection on the appropriate synthetic selection medium, cells were plates a second time on the same selection medium, before being plated and kept on YD plates. For fluorimeter measurements, cells were grown in Low Fluorescence synthetic medium (0.69% yeast nitrogen base without amino acids, riboflavin and folic acid (ForMedium), 2% glucose, all amino acids except the one used as a selection marker for plasmid maintenance, 50 mM MES, pH 5.0, filter-sterilized).

### Fluorescence microscopy and co-localization measurements

Pictures were obtained using a Zeiss Axio Observer 7 inverted epifluorescence microscope with a 100x oil immersion objective (Alpha Plan-Apochromat 100×/1.46 Oil) and were taken with Hamamatsu ORCA-Flash 4.0 LT sCMOS camera driven by Zen2.3 Pro software. Yeast cells were grown in synthetic medium and imaged in mid-log phase (OD_600_ = 2.0–4.0; an OD_600_ of 1.0 corresponds to approximately 10^7^ cells/ml). For imaging, they were directly mounted on a glass slide coated with a thin layer of agarose 1.5%. For co-localization measurements with the Golgi markers, cells were fixed with a solution of paraformaldehyde 4% in PBS during 20 min at 28 °C, then washed twice with 100 µl of PBS and mounted for imaging. For endosomes detection, FM4-64 was used^61^. To do so, 1 ml of culture was collected by centrifugation and resuspended in 100 µl of fresh YD and 1 µl of FM4-64 16 mM was added to the cell suspension. Cells were incubated during 20 minutes at 28 °C, then mounted for imaging. Alternatively, cells stained with FM4-64 were fixed prior to imaging, to be consistent with the quantification performed with the Golgi markers. In this case, 1 ml of culture collected by centrifugation was resuspended in 100 µl of YD at 4 °C and 2 µl of FM4-64 16 mM was added to the cell suspension. Cells were then incubated for 20 min on ice to allow FM4-64 to accumulate in the plasma membrane. They were then collected by centrifugation, resuspended in 100 µl of PBS and incubated for 5 min at 30 °C for FM4-64 to be internalized in endosomes. Cells were collected again and washed with 100 µl of PBS at 4 °C. Finally, cells were fixed with a solution of paraformaldehyde 4% in PBS during 20 min on ice, then washed twice with 50 µl of PBS at 4 °C and mounted for imaging that was performed in the following hour. Pictures were analyzed with Fiji/ImageJ 2.0.0-rc-69. They were cropped to only consider cells expressing the Golgi/endosomes markers. To quantify the co-localization, an object-based method based on the centers of mass – particle coincidence was performed using the JACoP plugin^62^. Prior to the plugin application, images were treated to identify the vesicles via the “Find maxima” tool, with the prominence value being manually adjusted for each picture, in order to get most of the visible vesicles and to avoid false positive dots. All the maxima were then resized to have a radius of 4 pixels and images generated in this way were then analyzed with the JACoP plugin. Representative images were background-subtracted with a rolling ball radius of 10–20 pixels and smoothed.

### Antibodies and western blotting

Routinely, protein samples were mixed with four time concentrated sample buffer (0.32 M Tris-HCl pH 6.8, 8% SDS, 40% glycerol, 0.02% bromophenol blue, 1% DTT), were separated on a 4–20% SDS/PAGE gel and Western Blotting analysis was carried out. Primary antibodies used in this study are rabbit anti-Sec22p (1:2,000; gift from C. Barlowe, Hanover, New Hampshire), rabbit anti-Prc1p (1:2,000; gift from H. Riezman, Geneva, Switzerland), anti-Pma1p (1:2,000; our laboratory^63^), rabbit polyclonal anti-Pmr1p (1:125; our laboratory^64^), polyclonal rabbit anti-GFP (1:1,000; Chromotek, PABG1), monoclonal rat anti-HA (1:1,000; Roche, 3F10). Secondary antibodies were horseradish peroxidase-coupled anti-rabbit IgG and anti-rat IgG secondary antibodies (1:10,000 dilution) and Lumi-Light Western Blotting Substrate was used (Roche Diagnostics). Chemiluminescence was captured using an Amersham Imager 600 (GE Healthcare) with automatic exposure time for high dynamic range.

### Subcellular fractionation

Subcellular fractionation was performed as in Demaegd *et al*.^56^, with slight modifications. The discontinuous sucrose gradient was prepared with 9 layers of 1.2 ml ranging from 22 to 54%, and 2.4 ml of the cell lysate was loaded at the top of the gradient. After ultracentrifugation, fractions of 1.2 ml were collected from the top of the gradient, were gently mixed with 300 µl of glycerol 50%, and were analyzed by immunoblotting or used for subsequent topology assay.

### Topology assay

Golgi enriched fractions obtained by subcellular fractionation were submitted to proteinase K digestion, with a protocol adapted from previous topology assay^65^. For this purpose, 15 µl of sample were incubated for 1 h at 30 °C in a final volume of 40 µl containing 1 µg/ml of proteinase K, 50 mM Tris, 1 mM CaCl_2_, pH 7.6. When needed for the negative control, Triton X-100 was added to a 1% (v/v) final concentration. For the control with inhibitors, the Golgi enriched fractions were pre-incubated for 10 min at room temperature with a protease inhibitor mixture (2.5 mM PMSF and a protease inhibitor cocktail with 16 µg/ml leupeptin, aprotinin, antipain, pepstatin, and chymostatin, final concentrations). At the end of the experiment, digestion was stopped by the addition of the protease inhibitor mixture to all the samples, then incubated for 10 min at room temperature, followed by the addition of 15 µl of sample buffer 4x concentrated preheated at 85 °C and incubated for an additional 10 min at 85 °C with mixing. The resulting samples were analyzed by SDS/PAGE and Western Blotting.

### Fluorimeter and *in vivo* pH calibration

Data were recorded using a JASCO FP8500 fluorimeter controlled by the Spectra Manager software. To convert the fluorescence measurements into pH values, *in vivo* calibration of the probe was performed. For this purpose, 100 ml of cell culture were grown in Low Fluorescence synthetic medium until OD_600_ = 3.0. Cells were collected by 3 min centrifugation at 1800 × g, were washed twice with 50 ml PBS and finally resuspended in 10 ml PBS supplemented with 0.16% digitonin. Cells were then incubated for 8 min at room temperature with mild agitation on a rocking table. Cells were collected again by 3 min centrifugation at 1800 × g, were resuspended in 10 ml PBS pre-cooled at 4 °C and were aliquoted by 1 ml fractions into 2 ml eppendorf tubes. Aliquots were centrifuged during 3 min at 4000 × g at 4 °C and cells were resuspended in 2 ml of the appropriate pH buffer (mix of citric acid 0.1 M – Na_2_HPO_4_ 0.2 M in adequate proportion). They were incubated for 10 min at room temperature, before being transferred into cuvettes for excitation spectra measurement. Emission intensity was measured at 507 nm during excitation from 360 to 490 nm (5 nm bandwidths, scan speed: 200 nm/min). The background fluorescence of cells transformed with an empty plasmid and treated in an identical way was subtracted from all these spectra, and the ratio of fluorescence emitted during excitation at 400 and 480 nm was plotted according to the pH. Finally, a sigmoidal four-parameter logistical curve was drawn through the experimental dots and used for subsequent determination of Golgi pH.

### Golgi and cytosol pH measurements at steady-state or during glucose pulse

Yeast cells expressing the Mnn2-HA-pHluorin** protein for Golgi pH measurements or the native pHluorin adapted to cytosolic pH measurements^29^ were grown at 28 °C in low Fluorescence synthetic medium. For Golgi pH measurements, 12 ml of cells were grown. When the OD_600_ reached 2.8–3.2, the appropriate volume to get 30 OD_600_ equivalent was collected by 5 min centrifugation at 1500 × g (swinging rotor). For steady-state pH measurements, cells were resuspended in 2 ml of fresh medium pre-warmed at 28 °C, the cell suspension was then transferred into a cuvette with a magnetic stirrer rotating at 300 rpm and fluorescence was measured in the fluorimeter set at 28 °C. The signal was recorded at 507 nm during excitation from 360 to 490 nm (5 nm bandwidths, scan speed: 200 nm/min). For glucose pulse measurements, cells collected were washed twice with 10 ml of low fluorescence synthetic medium without glucose pre-cooled at 4 °C and finally resuspended in 10 ml of the same medium. They were then incubated on ice during 30 min to starve them of glucose. After this starvation period, they were collected by centrifugation, resuspended in 2 ml of low fluorescence synthetic medium without glucose pre-warmed at 28 °C and incubated during 5 min in a water-bath at 28 °C. Then, 1.8 ml was transferred into a cuvette with a magnetic stirrer rotating at 300 rpm and fluorescence was measured in the fluorimeter set at 28 °C. The signal was recorded at 507 nm during alternate excitation at 400 and 480 nm. After 120 sec of recording, 200 µl of glucose 20% was added in the cuvette and the measurement was continued up to 10 min. For cytosolic pH measurements, the same protocol was used, except that 5 ml of cells were grown and collected by centrifugation, and that cells were resuspended every time in 5 ml of fresh medium. For all experiments, background fluorescence of cells transformed with an empty vector and treated similarly was also measured and subtracted as a blank from raw data. The 400/480 nm excitation ratio was finally converted into pH value.

### Statistical analyses

All statistical analyses were performed using GraphPad Prism version 8.2.0 for Windows (Graphpad Software, San Diego California USA). When possible, data sets were tested for Gaussian distribution with Kolmogorov–Smirnov test (α = 0.05). All data sets analyzed successfully passed normality test. Then, parametric tests were carried on: two-tailed t-test for the comparison of the means if there were only two conditions to compare, parametric one-way ANOVA with a Tukey test if there were more than two data groups to compare. Significance of means comparison is represented on the graphs with asterisks: *:p < 0.05; **:p < 0.01; ***:p < 0.001.

## Supplementary information

Supplementary Infomation.
