# Supplementary figures and images for: A new pH sensor localized in the Golgi apparatus of Saccharomyces cerevisiae reveals unexpected roles of Vph1p and Stv1p isoforms
Source: Sci Rep. 2020 Feb 5;10:1881. doi: 10.1038/s41598-020-58795-w (PMC7002768; doi:10.1038/s41598-020-58795-w)

Figure 2 (A)

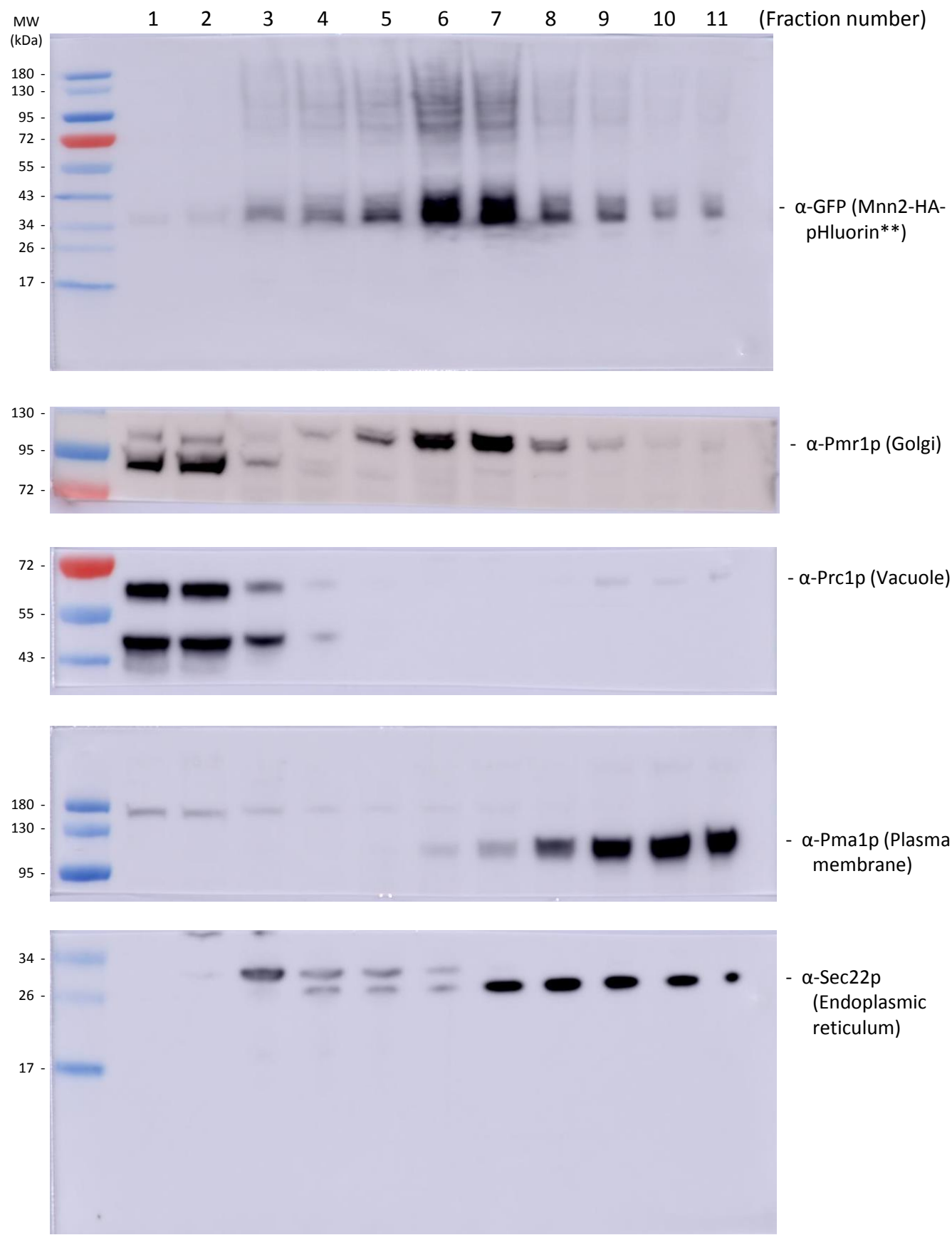

Figure 2 (C)

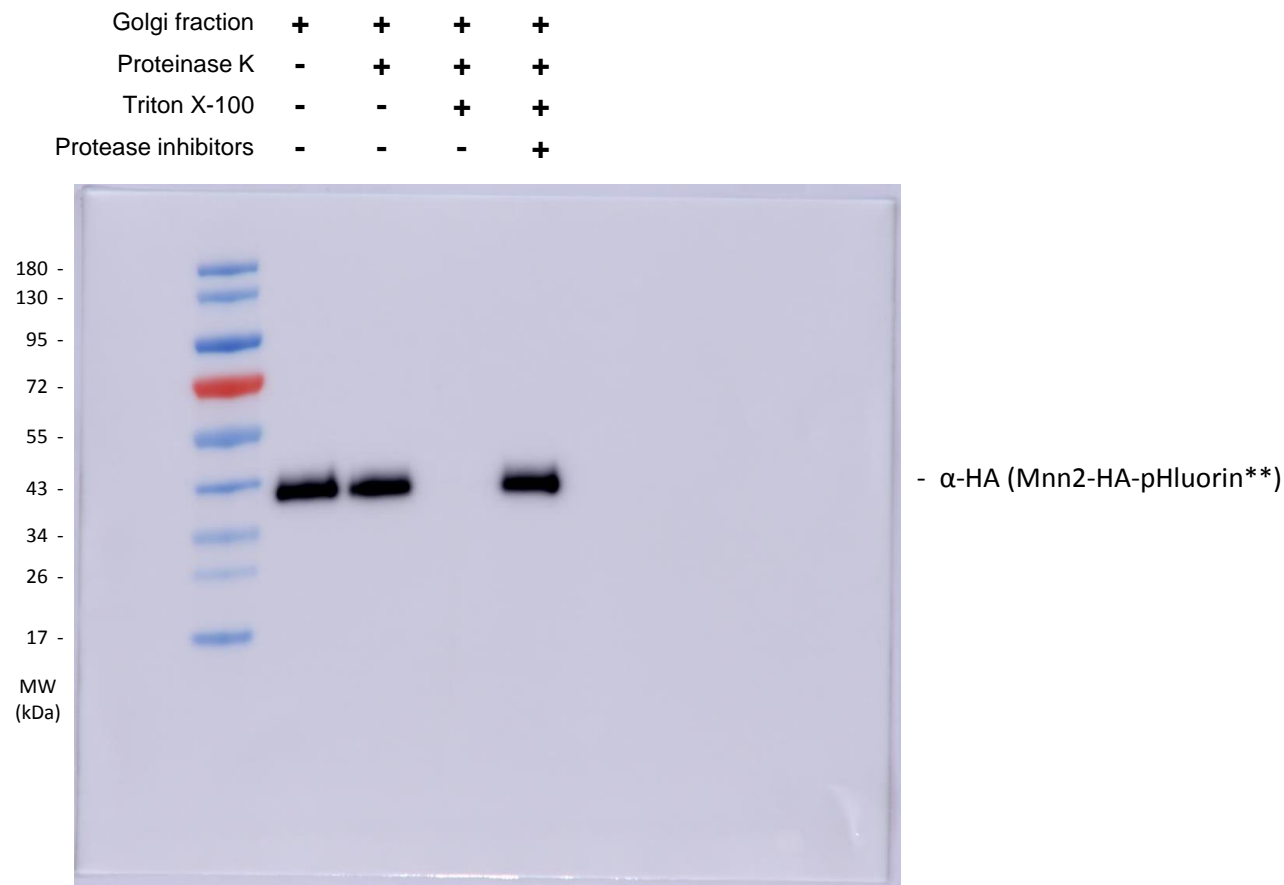

Supplement: Supplementary file 1 — Supplementary Infomation. [file 41598_2020_58795_MOESM1_ESM.pdf]
